# Supplementary material for: A computational framework to predict the spreading of Alzheimer’s disease
Source: Eng Comput. 2026 Apr 2;42(2):78. doi: 10.1007/s00366-026-02313-5 (PMC13043544; doi:10.1007/s00366-026-02313-5)
Supplement: Supplementary file 1 — (pdf 698 KB) [file 366_2026_2313_MOESM1_ESM.pdf]

Supplementary Material
A computational framework to predict the spreading of Alzheimer's disease

Ana Vazquez-Palomo[a], Covadonga Betegón[a], Johannes Weickenmeier[b], Emilio Martínez-Pañeda[b,*]

[a]*Department of Construction and Manufacturing Engineering, University of Oviedo, Gijón 33203, Spain*
[b]*Department of Engineering Science, University of Oxford, Oxford OX1 3PJ, UK*

Contents

1 Additional details of the theory **2**
1.1 Derivation of the governing equations from the weak form 2
1.2 Transformation from the material to the spatial transport equation 3
1.3 Mobility coefficient 3
1.4 Heterodimer formulation for tau and its coupling with $A\beta$ 4

2 Details of the COMSOL implementation **5**
2.1 Protein spread implementation 6
2.1.1 Built-in Transport Modelling 6
2.2 Cerebral atrophy implementation 7
2.2.1 User-defined Domain ODE 7
2.2.2 Built-in solid mechanics modelling 7

3 Additional subject information **8**
3.1 Subject dataset 8
3.2 Relative changes in regional brain volumes 9
3.3 Subject-specific analysis 10

*Corresponding author.
Email address: emilio.martinez-paneda@eng.ox.ac.uk (Emilio Martínez-Pañeda)

1. Additional details of the theory

1.1. Derivation of the governing equations from the weak form

Consider the Virtual Work provided in Section 2.2 of the main manuscript, particularised for simplicity to a single protein species,

$$\int_{\mathcal{B}_0} \Big(\boldsymbol{P} : \nabla\left(\delta\dot{\boldsymbol{\varphi}}\right) + \left[(\dot{c} - R)\, \delta\dot{\eta} - \mathbf{Q}{\cdot}\nabla(\delta\dot{\eta}) \right] \Big)\, \mathrm{d}V = \int_{\mathcal{B}_0} \mathbf{B}\cdot\delta\dot{\boldsymbol{\varphi}}\, \mathrm{d}V + \int_{\partial\mathcal{B}_0} \bar{\mathbf{T}}\cdot\delta\dot{\boldsymbol{\varphi}}\, \mathrm{d}S - \int_{\partial\mathcal{B}_0} \bar{q}\, \delta\dot{\eta}\, \mathrm{d}S. \quad (1)$$

Let us focus first on the mechanical terms,

$$\int_{\mathcal{B}_0} \boldsymbol{P} : \nabla(\delta\dot{\boldsymbol{\varphi}})\, \mathrm{d}V = \int_{\mathcal{B}_0} \mathbf{B}\cdot\delta\dot{\boldsymbol{\varphi}}\, \mathrm{d}V + \int_{\partial\mathcal{B}_0} \bar{\mathbf{T}}\cdot\delta\dot{\boldsymbol{\varphi}}\, \mathrm{d}S. \quad (2)$$

Now apply the product rule ($f(x)g'(x) = [f(x)g(x)]' - f'(x)g(x)$) to the first term,

$$\int_{\mathcal{B}_0} \boldsymbol{P} : \nabla(\delta\dot{\boldsymbol{\varphi}})\, \mathrm{d}V = \int_{\mathcal{B}_0} \nabla\cdot\left[\boldsymbol{P} : (\delta\dot{\boldsymbol{\varphi}})\right]\, \mathrm{d}V - \int_{\mathcal{B}_0} (\nabla\cdot\boldsymbol{P})\cdot\delta\dot{\boldsymbol{\varphi}}\, \mathrm{d}V. \quad (3)$$

Gauss divergence theorem ($\int_V \nabla\cdot\mathbf{A}\, \mathrm{d}V = \int_S \mathbf{A}\cdot\mathbf{N}\, \mathrm{d}S$) allows us to convert the first term of the right-hand side of Eq. (3) into a surface integral:

$$\int_{\mathcal{B}_0} \nabla\cdot\left[\boldsymbol{P} : (\delta\dot{\boldsymbol{\varphi}})\right]\, \mathrm{d}V = \int_{\partial\mathcal{B}_0} (\boldsymbol{P}\cdot\mathbf{N})\cdot\delta\dot{\boldsymbol{\varphi}}\, \mathrm{d}S. \quad (4)$$

And therefore,

$$\int_{\mathcal{B}_0} \boldsymbol{P} : \nabla(\delta\dot{\boldsymbol{\varphi}})\, \mathrm{d}V = \int_{\partial\mathcal{B}_0} (\boldsymbol{P}\cdot\mathbf{N})\cdot\delta\dot{\boldsymbol{\varphi}}\, \mathrm{d}S - \int_{\mathcal{B}_0} (\nabla\cdot\boldsymbol{P})\cdot\delta\dot{\boldsymbol{\varphi}}\, \mathrm{d}V. \quad (5)$$

Substitute (5) into (2):

$$\int_{\partial\mathcal{B}_0} (\boldsymbol{P}\cdot\mathbf{N})\cdot\delta\dot{\boldsymbol{\varphi}}\, \mathrm{d}S - \int_{\mathcal{B}_0} (\nabla\cdot\boldsymbol{P})\cdot\delta\dot{\boldsymbol{\varphi}}\, \mathrm{d}V = \int_{\mathcal{B}_0} \mathbf{B}\cdot\delta\dot{\boldsymbol{\varphi}}\, \mathrm{d}V + \int_{\partial\mathcal{B}_0} \bar{\mathbf{T}}\cdot\delta\dot{\boldsymbol{\varphi}}\, \mathrm{d}S. \quad (6)$$

Collect boundary terms and volume terms:

$$\int_{\mathcal{B}_0} \big(\nabla\cdot\boldsymbol{P} + \mathbf{B}\big)\cdot\delta\dot{\boldsymbol{\varphi}}\, \mathrm{d}V = \int_{\partial\mathcal{B}_0} \big(\boldsymbol{P}\cdot\mathbf{N} - \bar{\mathbf{T}}\big)\cdot\delta\dot{\boldsymbol{\varphi}}\, \mathrm{d}S. \quad (7)$$

Since this relation must hold for all $\delta\dot{\boldsymbol{\varphi}}$, then it follows from the fundamental theorem of variational calculus that each bracketed term of the above equation must vanish pointwise within their respective domains, giving us the strong mechanical balance and associated natural boundary condition:

$$\nabla\cdot\boldsymbol{P} + \mathbf{B} = \mathbf{0} \quad \text{in } \mathcal{B}_0, \quad (8)$$

$$\boldsymbol{P}\cdot\mathbf{N} = \bar{\mathbf{T}} \quad \text{on } \partial\mathcal{B}_0.. \quad (9)$$

We now treat the chemical (transport) piece, focusing for simplicity on one representative protein:

$$\int_{\mathcal{B}_0} (\dot{c} - R)\, \delta\dot{\eta} - \mathbf{Q}\cdot\nabla(\delta\dot{\eta})\, \mathrm{d}V = -\int_{\partial\mathcal{B}_0} \bar{q}\, \delta\dot{\eta}\, \mathrm{d}S. \tag{10}$$

Applying the product rule and Gauss divergence theorem to the $\mathbf{Q}$ term:

$$\int_{\mathcal{B}_0} \mathbf{Q}\nabla(\delta\dot{\eta})\, \mathrm{d}V = \int_{\mathcal{B}_0} \nabla\cdot[\mathbf{Q}\cdot(\delta\dot{\eta})]\; \mathrm{d}V - \int_{\mathcal{B}_0} (\nabla\cdot\mathbf{Q})\cdot\delta\dot{\eta}\, \mathrm{d}V = \int_{\partial\mathcal{B}_0} (\mathbf{Q}\cdot\mathbf{N})\cdot\delta\dot{\eta}\; \mathrm{d}S - \int_{\mathcal{B}_0} (\nabla\cdot\mathbf{Q})\cdot\delta\dot{\eta}\, \mathrm{d}V. \tag{11}$$

Now substitute (11) into (10), collecting boundary and volume terms,

$$\int_{\mathcal{B}_0} (\dot{c} - R + \nabla\cdot\mathbf{Q})\; \delta\dot{\eta}\, \mathrm{d}V = \int_{\partial\mathcal{B}_0} (\mathbf{Q}\cdot\mathbf{N} - \bar{q})\; \delta\dot{\eta}\, \mathrm{d}S. \tag{12}$$

And, as in the mechanical problem, the strong form equation and the associated natural boundary condition can be obtained by considering the fundamental theorem of variational calculus and noting that the relation above must hold for all $\delta\dot{\eta}$; rendering,

$$\dot{c} + \nabla\cdot\mathbf{Q} = R \quad \text{in } \mathcal{B}_0, \tag{13}$$

$$\mathbf{Q}\cdot\mathbf{N} = \bar{q} \quad \text{on } \partial\mathcal{B}_0. \tag{14}$$

1.2. Transformation from the material to the spatial transport equation

We choose to work with the concentration per unit reference volume $c(\mathbf{X}, t)$ in both the spatial and material frames, as in Schäfer et al. [1]. Conservation of mass implies the standard Piola transformation

$$\mathbf{Q} = J\,\mathbf{F}^{-1}\mathbf{q}, \qquad R = J\,r. \tag{15}$$

Let us now derive the spatial balance for the referential concentration, upon the assumption that convective transport is negligible relative to diffusion. Applying the Piola identity $\mathrm{Div}(J\boldsymbol{F}^{-1}\mathbf{q}) = J(\mathrm{div}\mathbf{q})\circ\varphi$ and substituting Eq. (15) into the strong form equation (13), one reaches,

$$\frac{\partial c}{\partial t} = -\mathrm{Div}_{\mathbf{X}}\mathbf{Q} + R = -J\,\mathrm{div}_{\mathbf{x}}\mathbf{q} + J\,r. \tag{16}$$

1.3. Mobility coefficient

Consider the definition of the chemical potential provided in the main manuscript

$$\mu_i = \frac{\partial\psi^c}{\partial c_i} = \mu_i^0 + RT\ln\left(\frac{c_i}{c_i^{\lim}}\right) \tag{17}$$

and the defined flux and mobility coefficients,

$$\mathbf{Q}_i = -\boldsymbol{M}_i \cdot \nabla_{\mathbf{X}}\, \mu_i = -\frac{c\boldsymbol{D}_i(\boldsymbol{F}, \vartheta)}{RT} \cdot \nabla_{\mathbf{X}}\, \mu_i \tag{18}$$

Then, noting that,

$$\nabla_{\mathbf{X}}\mu_i = RT\nabla_{\mathbf{X}}\left(\ln c_i\right) = \frac{RT}{c_i}\nabla_{\mathbf{X}}c_i \tag{19}$$

The flux can be derived as,

$$\mathbf{Q}_i = -\mathbf{M}_i \cdot \nabla_{\mathbf{X}}\mu_i = -\frac{c_i\boldsymbol{D}_i}{RT} \cdot \frac{RT}{c_i}\nabla_{\mathbf{X}}c_i = -\boldsymbol{D}_i\nabla_{\mathbf{X}}c_i \tag{20}$$

1.4. Heterodimer formulation for tau and its coupling with Aβ

Following the heterodimer kinetic model proposed by Pal and Melnik [2], we derive the source term governing tau pathology while explicitly accounting for the interaction between tau and amyloid-β proteins. This is done under a set of simplifying assumptions introduced by Schäfer et al. [1], under which the heterodimer formulation can be reduced to a Fisher-Kolmogorov-type equation. The amount of healthy tau protein $\tilde{c}_{\text{tau}}$ is governed by a production term (with rate constant $\tilde{k}_0$), a clearance term (with rate constant $\tilde{k}_1$), and two conversion mechanisms: a direct conversion into misfolded tau (with rate constant $\tilde{k}_{12}$), and an amyloid-β driven conversion controlled by the coupling coefficient $\tilde{k}_3$. The evolution of the misfolded tau population c_{tau} additionally involves a clearance term with rate constant k_1. The resulting heterodimer system reads

$$\frac{\partial \tilde{c}_{\text{tau}}}{\partial t} = \tilde{k}_0 - \tilde{k}_1\tilde{c}_{\text{tau}} - \tilde{k}_3 c_{\text{tau}}\tilde{c}_{\text{tau}}c_{\text{A}\beta} - \tilde{k}_{12}c_{\text{tau}}\tilde{c}_{\text{tau}}, \tag{21}$$

$$\frac{\partial c_{\text{tau}}}{\partial t} = -k_1 c_{\text{tau}} + \tilde{k}_3 c_{\text{tau}}\tilde{c}_{\text{tau}}c_{\text{A}\beta} + \tilde{k}_{12}c_{\text{tau}}\tilde{c}_{\text{tau}}. \tag{22}$$

Following Schäfer et al. [1], we assume that the amount of healthy tau is much larger than that of misfolded tau ($\tilde{c}_{\text{tau}} \gg c_{\text{tau}}$), such that the healthy population evolves on a much slower time scale and $\partial \tilde{c}_{\text{tau}}/\partial t \approx 0$. Solving Eq. (21) under this quasi-steady assumption yields

$$\tilde{c}_{\text{tau}} = \frac{\tilde{k}_0}{\tilde{k}_1 + c_{\text{tau}}(\tilde{k}_3 c_{\text{A}\beta} + \tilde{k}_{12})}. \tag{23}$$

This expression can be further approximated by a first-order Taylor expansion evaluated at $(\tilde{k}_3 c_{\text{A}\beta} + \tilde{k}_{12})c_{\text{tau}}/\tilde{k}_1 = 0$, rendering

$$\tilde{c}_{\text{tau}} \approx \frac{\tilde{k}_0}{\tilde{k}_1}\left(1 - \frac{\tilde{k}_3 c_{\text{A}\beta} + \tilde{k}_{12}}{\tilde{k}_1}c_{\text{tau}}\right). \tag{24}$$

Substituting Eq. (24) into Eq. (22) leads to a logistic-type evolution equation for the misfolded tau population,

$$\frac{\partial c_{\text{tau}}}{\partial t} = \left(\frac{\tilde{k}_0}{\tilde{k}_1}(\tilde{k}_3 c_{\text{A}\beta} + \tilde{k}_{12}) - k_1 \right) c_{\text{tau}} - \frac{\tilde{k}_0}{\tilde{k}_1^2}(\tilde{k}_3 c_{\text{A}\beta} + \tilde{k}_{12})^2 {c_{\text{tau}}}^2. \tag{25}$$

The maximum amount of misfolded tau, $c_{\text{tau}}^{\text{lim}}$, is obtained by enforcing $\partial c_{\text{tau}}/\partial t = 0$, yielding

$$c_{\text{tau}}^{\text{lim}} = \frac{\tilde{k}_1}{\tilde{k}_3 c_{\text{A}\beta} + \tilde{k}_{12}} \left(1 - \frac{k_1 \tilde{k}_1}{\tilde{k}_0(\tilde{k}_3 c_{\text{A}\beta} + \tilde{k}_{12})} \right). \tag{26}$$

We next rewrite Eq. (25) in terms of the normalized concentration of toxic tau,

$$\bar{c}_{\text{tau}} = \frac{c_{\text{tau}}}{c_{\text{tau}}^{\text{lim}}}, \tag{27}$$

such that its time evolution reads

$$\frac{\partial \bar{c}_{\text{tau}}}{\partial t} = \frac{1}{c_{\text{tau}}^{\text{lim}}} \frac{\partial c_{\text{tau}}}{\partial t}. \tag{28}$$

Substitution into the logistic equation yields the Fisher–Kolmogorov form

$$\frac{\partial \bar{c}_{\text{tau}}}{\partial t} = \alpha_{\text{tau}}\, \bar{c}_{\text{tau}} \left(1 - \bar{c}_{\text{tau}}\right), \tag{29}$$

with the effective tau growth-rate parameter

$$\alpha_{\text{tau}} = \frac{\tilde{k}_0}{\tilde{k}_1} \left(\tilde{k}_3 c_{\text{A}\beta} + \tilde{k}_{12} \right) - k_1. \tag{30}$$

As discussed in the main text, the amyloid-β concentration can itself be expressed in normalized form as

$$\bar{c}_{\text{A}\beta} = \frac{c_{\text{A}\beta}}{c_{\text{A}\beta}^{\text{lim}}}, \qquad c_{\text{A}\beta}^{\text{lim}} = \frac{\tilde{a}_1}{\tilde{a}_{12}} \left(1 - \frac{\tilde{a}_1 a_1}{\tilde{a}_{12} \tilde{a}_0} \right). \tag{31}$$

Substituting this expression into Eq. (30) renders the tau growth-rate explicitly dependent on the normalized amyloid-β concentration,

$$\alpha_{\text{tau}} \left(\bar{c}_{\text{A}\beta} \right) = \frac{\tilde{k}_0}{\tilde{k}_1} \left[\tilde{k}_3 \bar{c}_{\text{A}\beta} \frac{\tilde{a}_1}{\tilde{a}_{12}} \left(1 - \frac{\tilde{a}_1 a_1}{\tilde{a}_0 \tilde{a}_{12}} \right) + \tilde{k}_{12} \right] - k_1. \tag{32}$$

2. Details of the COMSOL implementation

We proceed to describe the numerical implementation in the finite element package `COMSOL` Multiphysics of the continuum models for protein spreading and cerebral atrophy. The primal fields and nodal degrees-of-freedom (DOFs) are the toxic tau relative concentration $\bar{c}_{\text{tau}}$, the toxic Aβ

relative concentration $\bar{c}_{A\beta}$, and the displacement components $\mathbf{u}$. The cerebral atrophy measure ϑ is also solved for, as an internal DOF. As described below, three `physics interfaces` are used in the implementation, two in-built ones (`Solid Mechanics` and `Transport in Solids`), and one user-defined domain ODE, implemented using the `Mathematics` module. Details can be found in the `COMSOL` documentation.

2.1. Protein spread implementation

2.1.1. Built-in Transport Modelling

We implement the spreading of toxic proteins using the `Transport in Solids` interface, which is part of the `Chemical Species Transport` module. Diffusion is considered to be the main transport mechanisms, and therefore convection is deactivated. We particularise the implementation to two species (`Dependent Variables`) with concentrations $\bar{c}_{\mathrm{tau}}$ and $\bar{c}_{A\beta}$. The transport equations are formulated in the spatial frame, which is explicitly selected in the interface settings. With this choice, the governing equation solved by COMSOL reads

$$\frac{\partial c_i}{\partial t} + \nabla \cdot \boldsymbol{\Gamma}_i = J s_i, \tag{33}$$

with the diffusive flux given by

$$\boldsymbol{\Gamma}_i = -\left(J\, \boldsymbol{F}^{-1} \boldsymbol{d}_i \boldsymbol{F}^{-T}\right) \nabla c_i. \tag{34}$$

Separate diffusion tensors are defined for tau and Aβ denoted d_{tau} and $d_{A\beta}$, following the expressions provided in the main text.

The axonal orientation field is imported into `COMSOL` through the `Definitions` node as an interpolation function in the white matter. This interpolated field defines the initial axonal unit vectors, denoted as $\mathbf{a}$, used in the tau diffusion tensor $\boldsymbol{d}_{\mathrm{tau}}$.

Reaction terms are implemented by adding a `Source` node for each species. In the source expressions s_{tau} and $s_{A\beta}$, we define the reaction terms corresponding to the model expressions R_{tau} and $R_{A\beta}$.

Zero-flux boundary conditions are enforced by selecting the default `No Flux` condition on the external boundary of the brain matter. Initial conditions are defined by prescribing a non-

zero concentration seed for each protein species within selected brain regions and setting the concentration to zero in the remaining domains.

2.2. Cerebral atrophy implementation

2.2.1. User-defined Domain ODE

The cerebral atrophy measure ϑ is implemented using the `Domain ODEs and DAEs` interface from the `Mathematics` module. The time evolution of ϑ is introduced through the damping term of the interface, with the damping coefficient set to unity.

$$\underbrace{\dot{\vartheta}}_{d_a \frac{\partial \vartheta}{\partial t}} = \underbrace{\left(1 + \frac{G_c}{G_0} \frac{1}{1 + \exp[-10^2 \, (c_{\text{tau}} - c_{\text{tau}}^{\text{crit}})]}\right) G_0}_{\text{Source term } f} . \tag{35}$$

The atrophy measure ϑ is initialized to represent a healthy state at the beginning of the simulation. Accordingly, the following initial conditions are imposed:

$$\vartheta(\mathbf{X}, 0) = 1, \qquad \partial_t \vartheta|_{t=0} = G_0. \tag{36}$$

2.2.2. Built-in solid mechanics modelling

The mechanical response of the brain tissue is implemented using the `Solid Mechanics` interface from the `Structural Mechanics` module. The displacement field $\mathbf{u}$ is defined as the dependent variable. A quasi-static formulation is selected by disabling inertial effects. With these settings, the balance of linear momentum solved by COMSOL reads

$$\nabla \cdot (\mathbf{F}\mathbf{S})^T = \mathbf{0}, \tag{37}$$

where $\mathbf{S}$ denotes the second Piola–Kirchhoff stress tensor.

A `Hyperelastic Material` node is added, and a compressible neo-Hookean formulation with a simplified volumetric strain energy (Simo–Pister) is selected. With this configuration, the strain energy density reads

$$W_s = \frac{G}{2} \left(tr(\boldsymbol{C}) - 3\right) - G \ln(J_e) + \frac{\lambda}{2} \left(\ln(J_e)\right)^2 . \tag{38}$$

where $\boldsymbol{C} = \boldsymbol{F}^{\mathsf{T}} \boldsymbol{F}$ is the right Cauchy-Green deformation tensor.

The neo-Hookean model is specified by two material parameters, namely the Lamé parameters λ and G. The corresponding energy density function W_s is equivalent to the pristine elastic

strain free energy density ψ_0^e in our formulation. To recover the atrophy-weighted elastic strain free energy density ψ^e, the Lamé parameters λ and G are multiplied by the atrophy measure ϑ. Since these parameters appear linearly in each term of the strain energy function, this modification results in $W_s = \vartheta \cdot \psi_0^e$.

Atrophy-induced deformation is introduced using the `External Strain` feature within the `Hyperelastic Material` node, where the atrophy deformation gradient $\mathbf{F}^a$ is prescribed.

Rigid body motion is prevented by applying a fixed constraint on the cerebrospinal fluid (CSF) domain. The displacement field is initialized by setting $\mathbf{u} = \mathbf{0}$ at the beginning of the simulation.

3. Additional subject information

3.1. Subject dataset

The data used in this study were obtained from the Alzheimer's Disease Neuroimaging Initiative (ADNI) database. Table 1 lists the subject IDs corresponding to the data used for the generation of each figure in the manuscript. The processed data used in the analysis are also provided alongside the code to facilitate reproducibility.

Table 1: Subject IDs used in each figure grouped by Alzheimer's disease (AD) and healthy controls (CN).

	**Figure**				
	[8] [9] [10] [11] [12] [14]	[13]		[15] [16]	
**Group**	AD	AD	CN	AD	CN
		006_s_689	002_S_0143		
		007_S_472	002_S_1280	006_S_6689	002_S_0413
		941_S_668	007_S_1222		
		003_S_122	007_S_5265		
		027_S_419	009_S_0751		
**Subject ID**	006_S_689	037_S_150	018_S_4400		
		037_S_241	033_S_0734		
		128_S_724	032_S_0677	941_S_6068	009_S_0751
		130_S_249	023_S_1190		
		135_S_489	021_S_0337		

3.2. Relative changes in regional brain volumes

Figure 1 extends the results presented in Section 4.3 (Brain deformations in healthy brain ageing and Alzheimer's disease) by reporting the temporal evolution of regional brain volumes in relative terms, for both healthy and diseased subjects in the analyzed cohort.

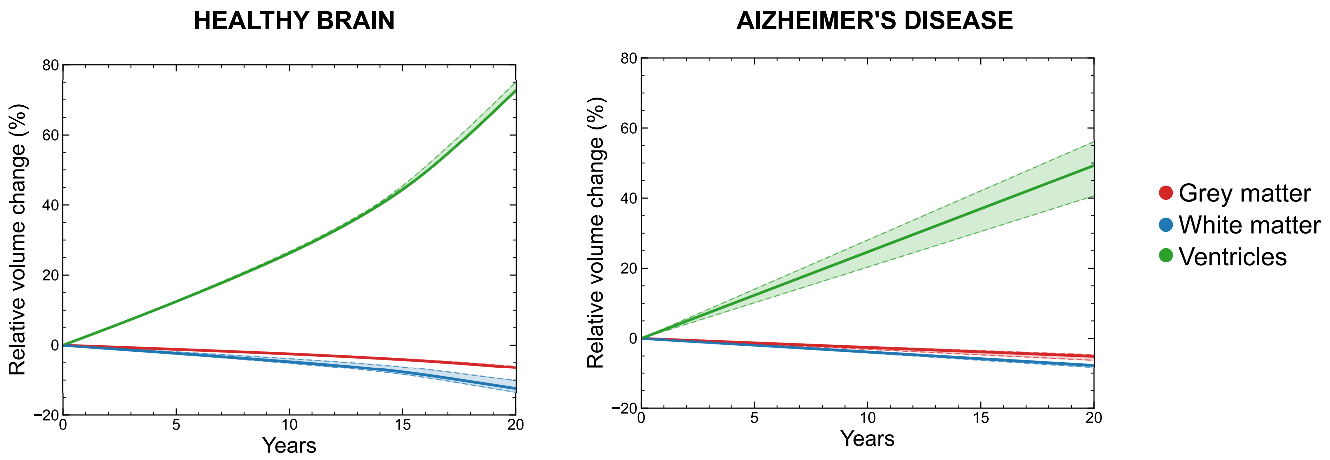

Figure 1: Relative change in regional brain volumes over a 20-year simulation period for a cohort of 10 healthy controls (left) and 10 subjects with Alzheimer's disease (right). Volumes are expressed as percentage change with respect to the initial value.

3.3. Subject-specific analysis

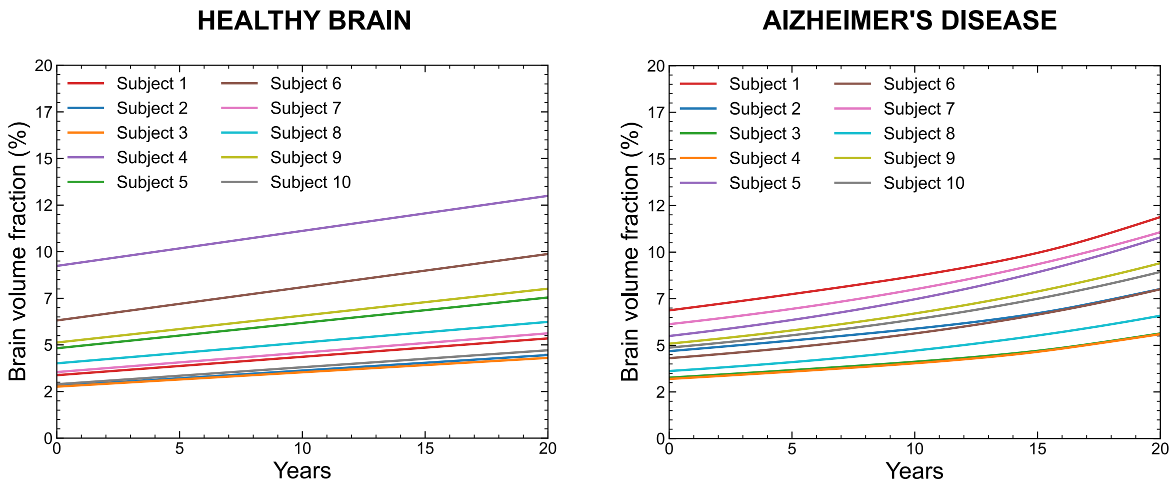

Figure 2: Subject-specific ventricular volume evolution over a 20-year simulation period for a cohort of 10 healthy controls (left) and 10 subjects with Alzheimer's disease (right). Volumes are expressed as percentages relative to the initial brain volume at $t = 0$.

The subject-specific ventricular volume evolution used to analyse the cohort trends discussed in Section 4.3 (Brain deformations in healthy brain ageing and Alzheimer's disease) is shown in Fig. 2.

References

[1] A. Schäfer, J. Weickenmeier, E. Kuhl, The interplay of biochemical and biomechanical degeneration in alzheimer's disease, Computer Methods in Applied Mechanics and Engineering 352 (2019) 369–388.

[2] S. Pal, R. Melnik, Nonlocal models in the analysis of brain neurodegenerative protein dynamics with application to alzheimer's disease, Scientific Reports 12 (2022) 7328.
